# Prevalence and associated factors of hypertension among adults in Gadarif in eastern Sudan: a community-based study

**DOI:** 10.1186/s12889-020-8386-5

**Published:** 2020-03-06

**Authors:** Saeed M. Omar, Imad R. Musa, Osman E. Osman, Ishag Adam

**Affiliations:** 1 Faculty of Medicine, Gadarif University, Gadarif, Sudan; 2 Royal Commission Hospital at AL Jubail Industrial City, Al Jubail, Kingdom of Saudi Arabia; 3 Faculty of Medicine, Alneelain University, Khartoum, Sudan; 4 Faculty of Medicine, University of Khartoum, P.O. Box 102, Khartoum, Sudan

**Keywords:** Hypertension, Blood pressure, Prevalence, Risk factors, Sudan

## Abstract

**Background:**

Hypertension is becoming an increasingly common health issue worldwide, especially in countries in Sub-Saharan Africa. Hypertension is the leading risk factor for premature death and disability worldwide, and it is the leading risk factor for mortality from cardiovascular diseases worldwide. The data on hypertension in Sudan that has been published is limited. We conducted this study to assess the prevalence of hypertension and its associated risk factors.

**Methods:**

A multistage sampling survey was conducted in Gadarif, Eastern Sudan, from January to May 2018 to investigate the prevalence of hypertension and associated factors in adults in Eastern Sudan. The World Health Organization (WHO) three-level stepwise approach questionnaire was used to gather sociodemographic characteristics (age, sex, height, weight marital status, education level, smoking habit, alcohol consumption habit, and family history of hypertension). Hypertension was defined as the presence of an average blood pressure of ≥140/90 mmHg or the reported use of anti-hypertensive medications for high blood pressure.

**Results:**

A total of 600 participants (mean age: 44.9 years) were enrolled in this study. Four hundred twenty-two (70.3%) participants were women, and 196 (32.7%) participants were obese. Of the 600 enrolled participants, 245 (40.8%) individuals had hypertension, 44 (7.3%) had been previously diagnosed with hypertension, and 201 (33.5%) were newly diagnosed with hypertension. A logistic regression analysis showed no significant associations across the education level, marital status, overweight and hypertension factors. However, an older age (adjusted OR = 3.20, 95% CI = 2.28–4.51, *P* < 0.001) and obesity (adjusted OR = 2.41, 95% CI = 1.57–3.69, P < 0.001) were associated with the presence of hypertension.

**Conclusion:**

There is a high rate of hypertension in Eastern Sudan, especially among older and obese individuals. Preventive measures, such as dietary measures, should be implemented.

## Background

It has been estimated that one out of four adults worldwide (1.39 billion people) have hypertension, and this rate is expected to increase as a result of various epidemiological and demographic factors, such urbanization, especially in low- and middle-income countries [1]. Approximately 74.7 million individuals have hypertension in Sub-Saharan Africa (SSA), and this number is expected to reach 125.5 million individuals by 2025 [2]. High blood pressure is the leading risk factor worldwide for premature death and disability measured in disability adjusted life years (DALYs) [3]. Hypertension is the leading risk factor worldwide for mortality from cardiovascular diseases, such as stroke and ischaemic heart disease [4, 5]. Various factors, such as an individual’s age [6, 7], smoking [8], consuming alcohol [9] and obesity [8, 10–12], were reported as factors associated with hypertension.

The prevalence of hypertension varies widely across countries and across regions within the same country. Thus, there is a need to assess the epidemiology (prevalence and risk factors) of hypertension in different settings. Investigating the epidemiology of hypertension is of paramount importance for health planners and academics, as well as physicians.

The epidemiology of hypertension (prevalence and associated risk factors) has been extensively investigated in many African countries, e.g., Ethiopia [9, 10], Tanzania [13], Uganda [14] and Madagascar [12], but little published data exist on the epidemiology of hypertension in Sudan [6, 7, 15]; in addition, there are no published data on the prevalence of and risk factors for hypertension in Eastern Sudan. The current study was conducted to investigate the prevalence and associated factors of hypertension in Gadarif in Eastern Sudan.

## Methods

### Study area

Gadarif is located on the Ethiopian and Eritrean borders. It is 400 km from the capital of Sudan (Khartoum). It is located at an altitude of 496 m above sea level between latitudes 14° and 16° north and longitudes 33° and 36° east. Gadarif has a population of 1,727,401 inhabitants.

### Study design and sampling

A multistage sampling survey was conducted in Gadarif, Eastern Sudan from January to May 2018. Initially, four localities (lowest administrative units in Sudan) were randomly selected from the 11 localities of Gadarif. Five districts within each locality were chosen using the systematic sampling method. Then, 150 subjects from 15 households from each district were selected based on the lottery method. The first member in each household who agreed to participate and met our inclusion criteria was elected. If the selected household was not inhabited or the inhabitants refused to participate, the next household was selected until the target number of subjects for the study was met. Two trained general practitioners were recruited to collect the data and perform the clinical examinations under the supervision of the primary investigator.

After signing an informed consent form, all adult (aged ≥ 18 years) Sudanese residents (including both the men and women) who were selected from households using a lottery method were enrolled in the study. Participants aged younger than 18 years, pregnant women, patients with poor cognitive functions and severely ill patients were excluded from this survey.

### Data collection

The World Health Organization (WHO) three-level stepwise approach questionnaire was used for data collection [16]. The questionnaire was used to gather data on the sociodemographic characteristics of the participants, including their age; sex; marital status, which was categorized as married, divorced or unmarried; education level (≤ secondary level or > secondary level); and family history of hypertension. In addition, smokers were identified as those “who had smoked more than 100 cigarettes in their lives and reported smoking in the past year” [17], and participants who consumed alcohol were identified as those who consumed “one or more drinks in the past month”.

### Procedures

Blood pressure was measured using an appropriate cuff size and a standard mercury sphygmomanometer after the participant rested for at least 10 min in a sitting position and with his or her arm resting at the level of the heart. With an appropriately sized cuff, the mean of two (at an interval of 1–2 min) blood pressure readings was calculated. If the difference between the two readings was > 5 mmHg, the blood pressure was measured again until the readings were consistent.

Patients were considered hypertensive if the reading for systolic BP was ≥140 mmHg, that for diastolic BP was ≥90 mmHg, or both criteria were met in both of the repeated measurements [18]. Each patient’s weight was measured in kilograms using (well-calibrated scales and adjusted to zero before each measurement) standard procedures; the person stood with minimal movement and his or her hands by his or her side. Moreover, the participants’ shoes and excess clothing were removed. Then, their heights were measured in centimetres after they stood straight with their backs against the wall and their feet placed side by side. The body mass index (BMI) was computed using the equation weight/height (m^2^). The BMI was categorized according to the WHO classification as underweight (< 18.5 kg/m^2^), normal weight (18.5–24.9 kg/m^2^), overweight (25.0–29.9 (kg/m^2^) or obese (≥30.0 kg/m^2^) [19]. Participants with a BP of > 140/90 mmHg were given a document to be followed in the nearest health centre. The data were gathered through a questionnaire that we developed for this study (Additional file 1).

The sample size of 600 individuals was selected based on previous studies on the prevalence of hypertension in Sudan [6, 7, 15], where 25.0% of participants were expected to have hypertension. The proportions of the main proposed risk factor (obesity) were expected to be 37.5% for hypertensive individuals and 25% for nonhypertensive individuals. It was calculated that a difference of 5% at α = 0.05 with a power of 80% can be detected with a sample size of 600 participants. It was assumed that 10% of the individuals might not respond or might have incomplete data.

### Statistical analysis

The software SPSS for Windows (version 20.0) was used to analyse the data. The chi-square test was used to compare the proportions between the participants who were diagnosed with hypertension and participants who did not have hypertension. A t-test and Mann-Whitney test were used to compare the continuous parametric and nonparametric data, respectively, between the two groups (hypertensive and nonhypertensive). Logistic regression analyses were performed with hypertension as the dependent variable and age, sex, marital status, education level, BMI and waist circumference as the independent variables. The independent variables were entered into the model if their univariate *P* value was < 0.20. Odds ratios (ORs) and 95% confidence intervals (CIs) were calculated, and a P value of less than 0.05 was considered statistically significant. Then, adjustments were performed using the backward likelihood ratio (LR) in the different models.

### Ethics

The study received ethical approval from the Research Board at the Faculty of Medicine at the University of Gadarif in Sudan. The reference number is 2016/39. Written informed consent was obtained from all enrolled patients.

## Results

### General characteristics

Seventy-eight households were approached, and the response rate for inclusion in the study was 96.1% (75 households). Six hundred participants were enrolled in the survey, and the mean and SD of the age of the participants was 44.9 and 16.5 years. Four hundred twenty-two (70.3%) participants were women, and the remaining 178 (29.7%) were men. The basic characteristics of the enrolled individuals are shown in Table 1.

**Table 1:** Socio-demographic characteristics of the enrolled individuals

Variable	Frequency	Proportion	Mean (95% CI) of the blood pressure
Marital status			
Married	425	70.8	97.4 (96.1–98.6)
Unmarried	132	22.0	92.3 (90.3–94.2)
Divorced/widowed	43	7.2	100.1 (96.4–103.7)
Education < secondary level	262	43.7	98.4 (96.7–100.0)
Smokers	15	2.5	94.0 (84.7–103.4)
Alcoholics	2	0.33	76.6 (34.3–119.0)
Family history (first-degree) of hypertension	174	29.0	95.6 (93.9–97.3)
Body mass index categories			
Underweight	44	7.3	89.8 (86.2–93.3)
Normal body mass index	201	33.5	94.5 (92.8–96.2)
Overweight	159	26.5	96.4 (94.5–98.4)
Obese	196	32.7	100.0 (98.3–101.7)

Of the 600 enrolled participants, 44 (7.3%) had been previously diagnosed with hypertension, and 201 (33.5%) were newly diagnosed with hypertension. Thus, in this survey, 245 (40.8%) individuals had hypertension (44 individuals were diagnosed previously, and 201 were newly diagnosed with hypertension).

The mean (95% CI) age [50.4 (48.4–52.3) years vs. 41.1 (39.4–42.8) years, *P* < 0.001] and the median (interquartile) of the BMI [28.6 (24.0–34.6) kg/m^2^ vs. 25.6 (21.7–29.7) kg/m^2^, P < 0.001] were significantly higher in the hypertensive individuals than in the nonhypertensive individuals.

There was no significant difference in the sex (*P* = 0.982), smoking (*P* = 0.606), alcohol consumption (*P* = 0.516) or family history of hypertension (*P* = 0.927) factors between the individuals with hypertension and those without hypertension. The patients with hypertension were more likely to be older, have a lower education level, be married and be obese (Table 2).

**Table 2:** Comparing the number (proportions) of the socio-demographic characteristics between hypertensive and non-hypertensive adults in eastern Sudan

Variables		Hypertensive (*n* = 245)	Non-hypertensive (*n* = 355)	*P*
Age	< the mean (44.9 years)	90 (36.7)	230 (64.8)	< 0.001
	≥ the mean (44.9 years)	155 (63.3)	125 (35.2)	
Sex	Male	72 (29.4)	106 (29.9)	0.982
	Female	173 (70.6)	249 (70.1)	
Education	< secondary level	125 (51.0)	137 (38.6)	0.003
	≥ secondary level	120 (49.0)	218 (61.4)	
Marital status	Married	193 (78.8)	232 (65.4)	< 0.001
	Unmarried	29 (11.8)	103 (29.0)	
	Divorced/widow	20 (5.6)	23 (9.4)	
Family history of hypertension	Yes	72 (29.4)	102 (28.7)	0.927
	No	173 (70.6)	253 (71.3)	
Smoking	Yes	5 (2.0)	10 (2.8)	0.606
	No	240 (98.0)	345 (97.2)	
Alcohol	Yes	0 (0)	2 (0.6)	0.516
	No	245 (100.0)	353 (99.4)	
Body mass index groups	Underweight	11 (4.5)	33 (9.3)	< 0.001
	Normal weight	65 (26.5)	136 (38.3)	
	Overweight	62 (25.3)	97 (27.3)	
	Obese	107 (43.7)	89 (25.1)	

The logistic regression analysis showed no significant associations across the education level, marital status, overweight or hypertension factors. However, an older age (adjusted OR = 3.20, 95% CI = 2.28–4.51, *P* < 0.001) and obesity (adjusted OR = 2.41, 95% CI = 1.57–3.69, *P* < 0.001) were associated with hypertension (Table 3).

**Table 3:** Logistic regression of the factors associated with hypertension in eastern Sudan

Variables		OR	95% CI	*P*
Age	< the mean(44.9 years)	Reference		< 0.001
	≥ the mean(44.9 years)*	3.20	2.28–4.51	
Education	≥secondary level	Reference		0.531
	<secondary level	1.12	0.79–1.93	
Marital status	Married	Reference		
	Unmarried	0.62	0.32–1.21	0.169
	Divorced/widow	0.91	0.43–1.93	0.813
Body mass index groups	Underweight	0.74	0.34–1.60	0.450
	Normal weight	Reference		
	Overweight	1.24	0.79–1.95	0.338
	Obese*	2.41	1.57–3.69	< 0.001

^*^Adjusted

## Discussion

The current study showed a high prevalence (40.8%) of hypertension in this region of Sudan. This result is consistent with the recent findings of one study, which reported that 39.6% of the village inhabitants in Northern Sudan had hypertension [20]. A similar rate (38.2%) of undiagnosed hypertension was reported in rural communities along the Nile River in Northern Sudan [6]. Approximately one-third (35.7%) of 954 individuals in Northern Sudan were found to have hypertension [7]. The high prevalence of hypertension in the different communities may be explained by improved statistics, increased awareness of hypertension [21], an increased prevalence of modern lifestyles that are associated with an increased prevalence of diabetes mellitus and obesity [22], and the widespread use of medication, such as steroids and non-steroidal anti-inflammatory drugs [23]. The prevalence rates of hypertension found in this study were much higher than those (15.9%) recently reported for four states (Khartoum, Gezira, Blue Nile, and Kassala) in Sudan [15]. Interestingly, a much higher prevalence (49.1%) of hypertension was reported among 513 adults in a rural setting in Madagascar [12].

Conversely, a lower rate (28.0%) of hypertension was reported in Tanzania [13] and in a population-based sectional study that enrolled 67,397 participants in Ethiopia (31.9%) [24]. In neighbouring Ethiopia, while a low rate (10.5%) of hypertension was reported in a hospital-based study [8], a prevalence rate of 34.7% for hypertension was reported in the capital, Addis Ababa [10]. In a meta-analysis of 43,025 older adults (> 53 years) in 15 African countries, it was reported that the overall pooled prevalence of hypertension was 57.0% (ranging from 22.3 to 90.0%) [25]. Differences in age, culture, dietary habits, behaviour, race and genetics in the different settings may explain the differences in the prevalence of hypertension in the various populations.

In the current study, an older age was associated with hypertension (adjusted OR = 3.20, 95% CI = 2.28–4.51). This result is consistent with the finding in a previous study, showing a significant association between an increasing age and the prevalence of hypertension in Northern Sudan [6]. A significant association between an increasing age and the prevalence of hypertension was reported in Northern Sudan [7]. Because of our study design (controlled for age), it might not be valid to compare our results (age and hypertension) with the results of the large study that was recently conducted in four states (Khartoum, Gezira, Blue Nile and Kassala) in Sudan [15]. Several studies have shown that an older age is significantly associated with hypertension in neighbouring countries, including Ethiopia [8, 9, 11], Tanzania [13] and Madagascar [12]. Similarly, a recent meta-analysis including 43,025 older adults (> 53 years) in 15 African countries showed that an older age is independently associated with hypertension [25]. The high prevalence of hypertension in elderly patients may be explained by the increased stiffness of the aorta and the other arteries as a result of the ageing process.

Our results showed that obese individuals had a 2.41 times higher risk for hypertension. A significant association between obesity and hypertension has been found in Northern Sudan [7]. Our finding showing an association between obesity and hypertension is consistent with the findings that were recently reported for four states (Khartoum, Gezira, Blue Nile and Kassala) in Sudan [15]. Several previous studies conducted in Africa—e.g., Uganda [14], Ethiopia [8, 10, 11] and Madagascar [12]—have reported significant associations between obesity and hypertension. In a recent meta-analysis of 43,025 adults in 15 African countries, overweight/obesity was shown to be independently associated with hypertension [25]. The association between obesity and hypertension may be explained by the increased plasma endothelin-1 and nitric oxide production and adiposity among obese individuals [26, 27]. Moreover, up to a 63% resolution of hypertension can be achieved by the reduction of one’s body weight [28, 29].

Our results and the results of a recent meta-analysis showed there is no association between an individual’s sex and the presence of hypertension [25]. In the current study, there were no significant associations between hypertension and the marital status, education level, alcohol consumption or smoking habit factors. No significant associations were found between hypertension and the marital status, education level, alcohol consumption or smoking habit factors in Uganda [14]. Previous studies have shown that smoking is associated with hypertension [8]. Interestingly, the rates for both smoking (2.5%) and alcohol consumption (0.33%) were low in our survey. Perhaps the rates of smoking and alcohol consumption were underestimated, as many individuals may not have been willing to reveal these habits.

### Limitations

It is worth mentioning that our results should be compared with the results of other studies with caution; the differences in the participant selection method used, size of the study population, and lifestyles and ethnicities of the participants should be considered. This study required only two readings, and the time for which the test administrators needed to visit the households may have affected the selection of the participants. Perhaps the predominance of females (70.3%) in the current study population might be explained by the idea that more males than females were working during the time of the survey, and many females were housewives. Some factors, e.g., physical inactivity [11], dietary factors [10] and high fasting blood glucose levels [9], which have reportedly been associated with hypertension, were not explored in our survey, which should be considered limitations of the study.

## Conclusion

There is a high prevalence of hypertension in Eastern Sudan, especially among older and obese individuals. Preventive measures, such as dietary measures, should be implemented.

## Supplementary information

**Additional file 1.** questionnaire for the prevalence and associated factors of hypertension among adults in Gadarif in Eastern Sudan: a community based study.

## Data Availability

The datasets used and/or analysed during the current study are available from the corresponding author on reasonable request.

## Abbreviations

BMI: Body mass index
BP: Blood pressure
CI: Confidence interval
ORs: Odds ratios
SD: Standard deviation
