# Supplementary material for: Prevalence and associated factors of hypertension among adults in Gadarif in eastern Sudan: a community-based study
Source: BMC Public Health. 2020 Mar 6;20:291. doi: 10.1186/s12889-020-8386-5 (PMC7059678; doi:10.1186/s12889-020-8386-5)
Supplement: Supplementary file 1 — Additional file 1. questionnaire for the prevalence and associated factors of hypertension among adults in Gadarif in Eastern Sudan: a community based study. [file 12889_2020_8386_MOESM1_ESM.docx]

**Flow chart for** **Epidemiology of hypertension at Gadarif state eastern Sudan**

Age male female

married - --- unmarried divorced/widow

Education Nill/traditional ------- secondary university and above

Profession unemployed civil servant private sector --- self retiredp

HT NO Yes Duration

DM NO Yes Duration

Diagnosed as hypertension before and not treated Yes No

Diagnosed as Diabetes before and not treated Yes No

**Other diseases:**

HT DM Thyroid IHD H Failure Renal disease CVA

Family history of HT in the first degree relatives NO Yes

Family history of DM in the first degree relatives NO Yes

Smoking NO --- yes Alcohol No Yes

Weight height BMI Waist circumference

Blood pressure1: systolic 1 diastolic 1

Blood pressure2: systolic2 diastolic 2
